# Particle Engineering by Nano Spray Drying: Optimization of Process Parameters with Hydroethanolic versus Aqueous Solutions

**DOI:** 10.3390/pharmaceutics14040800

**Published:** 2022-04-06

**Authors:** Khaled Almansour, Raisuddin Ali, Fawaz Alheibshy, Tariq J. Almutairi, Rakan F. Alshammari, Nasser Alhajj, Cordin Arpagaus, Mustafa M.A. Elsayed

**Affiliations:** 1 Department of Pharmaceutics, College of Pharmacy, University of Hail, Hail 55473, Saudi Arabia; kh.almansour@uoh.edu.sa (K.A.); fa.alheibshy@uoh.edu.sa (F.A.); tariq.almutairi@outlook.sa (T.J.A.); rakan.faisalal@gmail.com (R.F.A.); 2 Department of Pharmaceutics, College of Pharmacy, King Saud University, Riyadh 12372, Saudi Arabia; ramohammad@ksu.edu.sa; 3 Pharmaceutical and Molecular Biotechnology Research Centre, Waterford Institute of Technology, X91 K0EK Waterford, Ireland; nasser.al-hajj@postgrad.wit.ie; 4 Institute for Energy Systems, Eastern Switzerland University of Applied Sciences, 9471 Buchs, Switzerland; cordin.arpagaus@ost.ch; 5 Department of Pharmaceutics, Faculty of Pharmacy, Alexandria University, Alexandria 21521, Egypt

**Keywords:** nano spray drying, particle engineering, critical process parameters, quality by design

## Abstract

Nano spray drying has emerged as an outstanding platform for engineering micro- and nanoparticles, with growing applications in various areas of drug delivery. As a new technology involving distinct technical design, parameters of the nano spray drying process are not fully elucidated. In a quality-by-design approach, the aim of the current study was to gain thorough understanding of critical determinants of product characteristics in the Büchi Nano Spray Dryer B-90. Following a factorial experimental design, a series of spray drying experiments were conducted to gain new insights into the influences of the inlet temperature, the spray solvent, and the solute concentration in the spray solution on the yield, the moisture content, and the particle size of the nano spray-dried powder material. Special consideration was given to the potential of using hydroethanolic in comparison with aqueous solvent systems and to particle engineering for pulmonary drug delivery. Lactose and mannitol, widely used as excipients in dry powder inhalation formulations, were used as model materials. Lactose and mannitol are known to spray dry in amorphous and crystalline forms, respectively. The yields of spray drying of lactose and mannitol amounted generally to 71.1 ± 6.6% *w*/*w* and 66.1 ± 3.5% *w*/*w*, respectively. The spray-dried materials exhibited generally a number-weighted median particle diameter of 1.6 ± 0.2 μm and a volume-weighted median particle diameter of 5.1 ± 1.0 μm. A detailed analysis of the results improved understanding of the interplay between process parameters in the Nano Spray Dryer. The results demonstrate that optimization of spray generation is the key to yield optimization. On the other hand, particle size is determined by the spray mesh pore size and the spray solution degree of saturation. Selection of an appropriate spray solvent and using spray solution additives could optimize spray flow. In parallel, the spray solvent and the solute concentration in the spray solution determine the degree of saturation. Guidance on optimization of particle engineering by nano spray drying is provided.

## 1. Introduction

**Figure 1:**
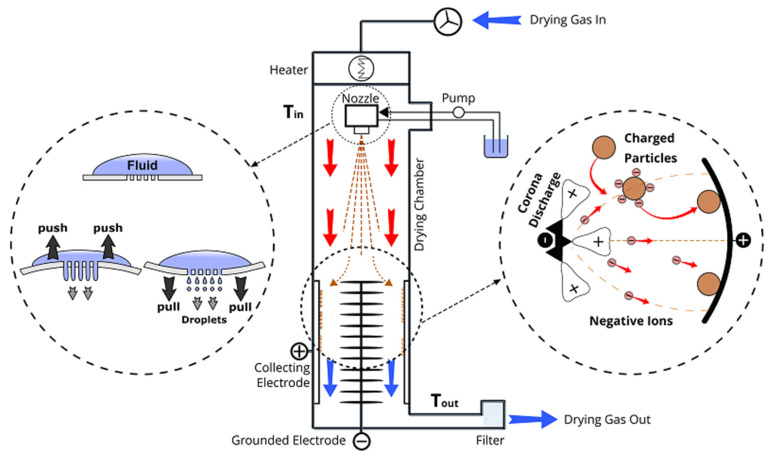
A schematic diagram of the Büchi Nano Spray Dryer B-90 and its characteristic elements (illustration adapted from the operation manual [13], with permission). The left and the right circles illustrate the functioning principles of the vibrating-mesh atomizer and the electrostatic particle collector, respectively. $T_{\mathrm{in}}$ and $T_{\mathrm{out}}$ are the inlet and the outlet temperatures.

Spray drying is well established as a technique for production of pharmaceutical powders [1,2,3,4]. Spray drying provides a flexible, controllable, and scalable approach for particle engineering. The Nano Spray Dryer B-90 (Büchi Labortechnik AG, Flawil, Switzerland) has emerged, since it was launched in 2009, as a platform for engineering of particles at the low-micron (<10 μm) and the submicron scale. The Nano Spray Dryer features three main differences to conventional spray dryers (Figure 1) [5,6]. First, spray generation in the Nano Spray Dryer takes place by a vibrating-mesh atomizer, whereas conventional spray dryers involve pressure, two-fluid, centrifugal, or ultrasound atomizers. Piezoelectric vibration of the spray mesh in the Nano Spray Dryer allows fast generation of precisely sized droplets. Second, particle collection in the Nano Spray Dryer occurs by electrostatic precipitation. A high-voltage electric field allows mass-independent collection of particles. It thereby allows more efficient recovery of small (<5 μm) particles than cyclones in conventional spray dryers. Third, the drying gas flow in the Nano Spray Dryer is generally laminar, which minimizes yield loss. The Nano Spray Dryer exhibited growing applications in various areas of pharmaceutical and particle engineering research. The applications are comprehensively reviewed in references [5,6,7]. Its particle size range and the precise control of particle size and morphology make it especially suitable for development of dry powder inhalation formulations [8,9,10,11,12]. As a lab-scale spray dryer, the amount of powder it produces per batch is in the order of a gram, limiting its applications to research studies. The first-generation Nano Spray Dryer B-90 was followed by the second-generation Nano Spray Dryer B-90 HP, in which spray generation and control are improved.

The technical differences between the Nano Spray Dryer and conventional spray dryers can alter the influences of process parameters on product characteristics. Concerning spray generation by the vibrating-mesh atomizer, the effects of the mesh pore size on the size of spray droplets or spray-dried particles have been studied by several investigators [11,12,14,15,16,17]. The influences of the inlet temperature in the Nano Spray Dryer on product characteristics, such as the yield, the moisture content, and the particle size, have been also studied by several investigators [11,12,14,16,17,18]. Longest et al. [19] have recently developed a computational fluid dynamics model of the Nano Spray Dryer B-90. The influences of the solute concentration in the spray solution on product characteristics, such as the yield and the particle size, have been explored as well [11,12,16,20]. Yet, some questions remain open. For example, the potential of organic solvent and cosolvent systems compared with aqueous systems has received little consideration in the research field.

Ethanol is safe for inhalation. It is widely used as a cosolvent in metered-dose inhaler formulations [21]. Compared with water, using an ethanol-water mixture as a spray solvent for nano spray drying has been reported to result in smaller particles [9,17]. The observation suggests a potential advantage of cosolvent systems in development of dry powder inhalation formulations by nano spray drying. However, the observation could not be elucidated.

The aim of the current study was to gain a deep, thorough understanding of critical determinants of product characteristics in the Nano Spray Dryer B-90, with particular interest in the potential of hydroethanolic versus aqueous solvent systems. The solvent systems provide different evaporation rates, viscosities, surface tensions, and solute solubilities. To this end, a series of spray drying experiments were conducted, following a factorial experimental design. Lactose and mannitol were used as model materials. Lactose and mannitol are widely used as excipients in dry powder inhalation formulations and are known to spray dry in amorphous and crystalline forms, respectively. In addition, the influences of the inlet temperature, the spray throughput, and the solute concentration in the spray solution on the yield, the moisture content, and the particle size of the nano spray-dried powder material were explored.

## 2. Materials and Methods

### 2.1. Materials

α-lactose monohydrate (Lactohale^®^ LH210) was kindly provided by DFE Pharma (Goch, Germany). D-Mannitol (ACS reagent) was from Sigma-Aldrich (Saint Louis, MO, USA). Physical properties of lactose and mannitol are presented in Table 1. Ethanol 96% *v/v* (AnalaR^®^ NORMAPUR^®^) was from VWR International S.A.S. (Fontenay-sous-Bois, France). Type 1 ultrapure water was produced by a Milli-Q^®^ Direct Water Purification System (Millipore, Molsheim, France).

### 2.2. Experimental Design

The experimental design is outlined in Table 2. With lactose, the influences of the inlet temperature at two levels (80 and 110 °C), the concentration of ethanol in the spray solution at three levels (0.0, 15.0, and 50.0 *w*/*w*), and the solute concentration in the spray solution at two levels (1.50 and 3.00 *w*/*w*) were studied. With aqueous lactose solutions, the experimental design was further extended to study the influences of the spray rate at three levels: low (<25 g/h), medium (25–45 g/h), and high (>45 g/h). This was not possible with hydroethanolic solutions since they exhibited a maximum spray rate of only 21 g/h (using the selected spray mesh, see Section 2.3). Randomly selected experiments were repeated. With mannitol, the influences of the inlet temperature at two levels (80 and 110 °C), the concentration of ethanol in the spray solution at two levels (0.0 and 50.0 *w*/*w*), and the solute concentration in the spray solution at two levels (1.50 and 3.00 *w*/*w*) were studied. Mannitol experiments were fewer than lactose experiments since they principally aimed to validate the findings of lactose experiments.

### 2.3. Spray Drying

Spray drying was conducted using a Büchi Nano Spray Dryer B-90 (Büchi Labortechnik AG, Flawil, Switzerland) with the tall drying chamber setup (chamber length ≈ 90 cm; drying, i.e., nozzle-to-collector, length ≈ 75 cm). A spray mesh with 5.5-μm-diameter pores was used for spray generation in all experiments. Regarding pulmonary drug delivery, respirable particles can be achieved using the 5.5-μm spray mesh [10]. It is yet associated with improved spray throughput compared to the 4.0-μm spray mesh. Lactose solutions exhibited steady spray flow. In contrast, mannitol solutions were associated with poor spray flow. Inspired by earlier experimental data [9,10], addition of hydrochloric acid at a low concentration was found to improve the spray flow for mannitol solutions (the observation is discussed in Section 3.1). Hydrochloric acid was thus added to a concentration of 0.02% *w*/*w* to all mannitol spray solutions. For aqueous lactose solutions, the spray head output was set to 50 to 100%, which produced spray rates of 14 to 60 g/h. This range allowed studying the influences of the spray rate on the nano spray drying process. For other experiments, the spray intensity was fixed as given in Table 2. The spray rate was not always reproducible. Therefore, we had to record the actual spray rate in each experiment. The drying gas was air dehumidified by a Büchi Dehumidifier B-296. The gas flow rate was set to 135 L/min. The nano spray drying process parameters were recorded using the Nano Spray Dryer Records 1.2 software.

Each spray drying experiment was designed to produce 2.0 g of powder. The nano spray-dried powder materials were collected manually with a scraper and then stored over silica gel in a desiccator at room temperature until further analysis.

### 2.4. Characterization of Spray Solutions

The viscosities of the spray solutions were measured using an A&D SV-10 tuning-fork Vibro viscometer (A&D Company, Limited, Tokyo, Japan) at 40 °C. The measurement technique requires correction for density. For this purpose, the densities of the solutions were measured using a 100 mL glass density bottle at 40 °C, after calibration with water at the same temperature.

### 2.5. Characterization of Nano Spray-Dried Powder Materials

#### 2.5.1. Moisture Content

The moisture contents of the nano spray-dried powder materials were measured in terms of the losses on drying using a Phoenix MOC-120H moisture balance (Phoenix Instrument, Garbsen, Germany). Drying was conducted at 105 °C. The measurements were set to end when the change in the moisture content over two consecutive 30-s periods falls below 0.05% *w*/*w*. It is noteworthy that the loss on drying cannot differentiate between residual moisture and residual ethanol. However, it is plausible to assume that the contribution of residual ethanol to the loss on drying here is minor, in comparison with residual water.

#### 2.5.2. Particle Morphology and Size

Particle morphology was studied by scanning electron microscopy (SEM). Samples of selected nano spray-dried powder materials were coated with gold using a Quorum Q150R S sputter coater (Quorum Technologies Ltd., Laughton, UK) at 20 mA for 90 s. The samples were then visualized using a ZEISS EVO LS 10 scanning electron microscope (Carl Zeiss Microscopy GmbH, Jena, Germany). Images were captured using the ZEISS SmartSEM version 5.05 software and analyzed using the JMicroVision version 1.3.4 software (Roduit, N., Geneva, Switzerland). For each of the selected nano spray-dried powder materials, the maximum Feret’s diameters of 300–900 particles, which were randomly selected from 2–3 images, were determined. Grubbs’s test was used at a significance level of 0.001 to determine outliers. After removing outliers, the number-weighted ($D_{N}$) and the volume-weighted ($D_{V}$) particle size distributions were constructed. The mean diameter ($D_{\mathrm{mean}}$), the 10th percentile ($D_{10}$) of the cumulative undersize particle diameter distribution, the median diameter ($D_{50}$), the 90th percentile ($D_{90}$) of the cumulative undersize particle diameter distribution, and the Span ($\mathrm{Span}=\left(D_{90}-D_{10}\right)/D_{50}$) were calculated.

#### 2.5.3. Thermal Analysis

Thermal analysis was conducted for selected nano spray-dried powder materials using a Shimadzu DSC-60 Plus differential scanning calorimeter equipped with a TA-60WS thermal analysis system and the TA-60WS version 2.21 software (Shimadzu Corporation, Kyoto, Japan). Accurately weighed samples of the powder materials were heated in aluminum pans under nitrogen purge from 30 °C to 250 °C at a heating rate of 5 °C/min.

### 2.6. Data Analysis

Mathematical and statistical data analysis was performed using OriginPro 2021 (OriginLab Corporation, Northampton, MA, USA). Presented data are means ± standard deviations, unless otherwise stated. Statistical comparisons were carried out using analysis of variance (ANOVA) with Tukey’s post hoc test. The significance level was 0.05, unless otherwise stated. For example, data of nano spray drying of aqueous lactose solutions were studied by three-way analysis of variance, takings the inlet temperature, the spray rate, and the concentration of lactose in the spray solution as independent variables. To study influences of the spray solvent, data of nano spray drying of aqueous solutions at low spray rates were compared to data of nano spray drying of hydroethanolic solutions by three-way analysis of variance, takings the inlet temperature, the concentration of ethanol in the spray solution, and the concentration of lactose in the spray solution as independent variables. Data of nano spray drying of aqueous and hydroethanolic solutions of mannitol were studied by three-way analysis of variance, takings the inlet temperature, the spray solvent, and the concentration of mannitol in the spray solution as independent variables.

## 3. Results and Discussion

Characteristics of nano spray-dried powder materials are summarized in Table 3. The yield of spray drying of lactose generally amounted to 71.1 ± 6.6% *w*/*w*, whereas the yield of spray drying of mannitol generally amounted to 66.1 ± 3.5% *w*/*w*. The yield here refers to the spray-dried powder material deposited on the surface of the collection electrode and is presented as a fraction of the amount of the solute dissolved in the spray solution.

**Figure 2:**
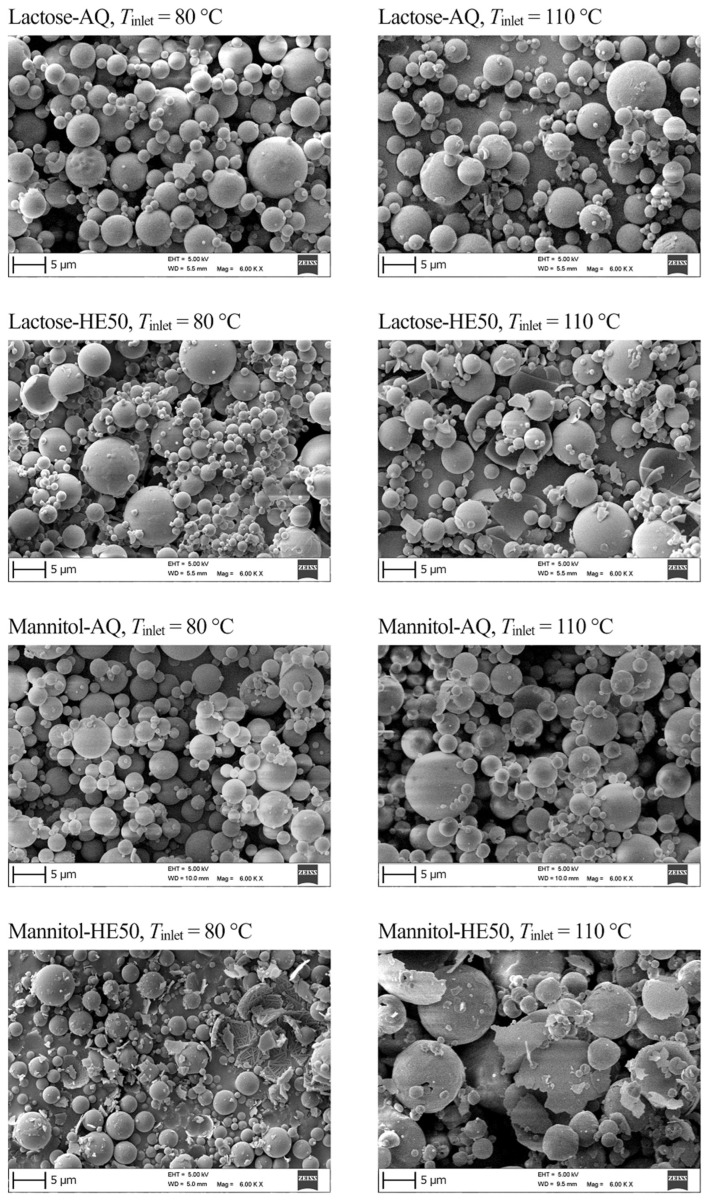
Scanning electron micrographs of nano spray-dried lactose and mannitol powder materials. Presented micrographs are for materials prepared by spray drying of aqueous (AQ) or hydroethanolic (HE50) solutions with a solute concentration of 1.50% *w*/*w* at an inlet temperature, $T_{\mathrm{inlet}}$, of 80 or 110 °C.

Scanning electron micrographs of selected nano spray-dried lactose and mannitol powder materials are presented in Figure 2. The particles were all spherical. For both lactose and mannitol, using a hydroethanolic spray solvent resulted, generally, in materials with remarkable amounts of fractured particles. Spray-dried lactose materials generally exhibited a number-weighted median particle diameter, $D_{N,50}$, of 1.6 ± 0.2 μm and a volume-weighted median particle diameter, $D_{V,50}$, of 4.8 ± 0.7 μm. Spray-dried mannitol materials generally exhibited a number-weighted median particle diameter, $D_{N,50}$, of 1.7 ± 0.3 μm and a volume-weighted median particle diameter, $D_{V,50}$, of 5.6 ± 1.3 μm.

**Figure 3:**
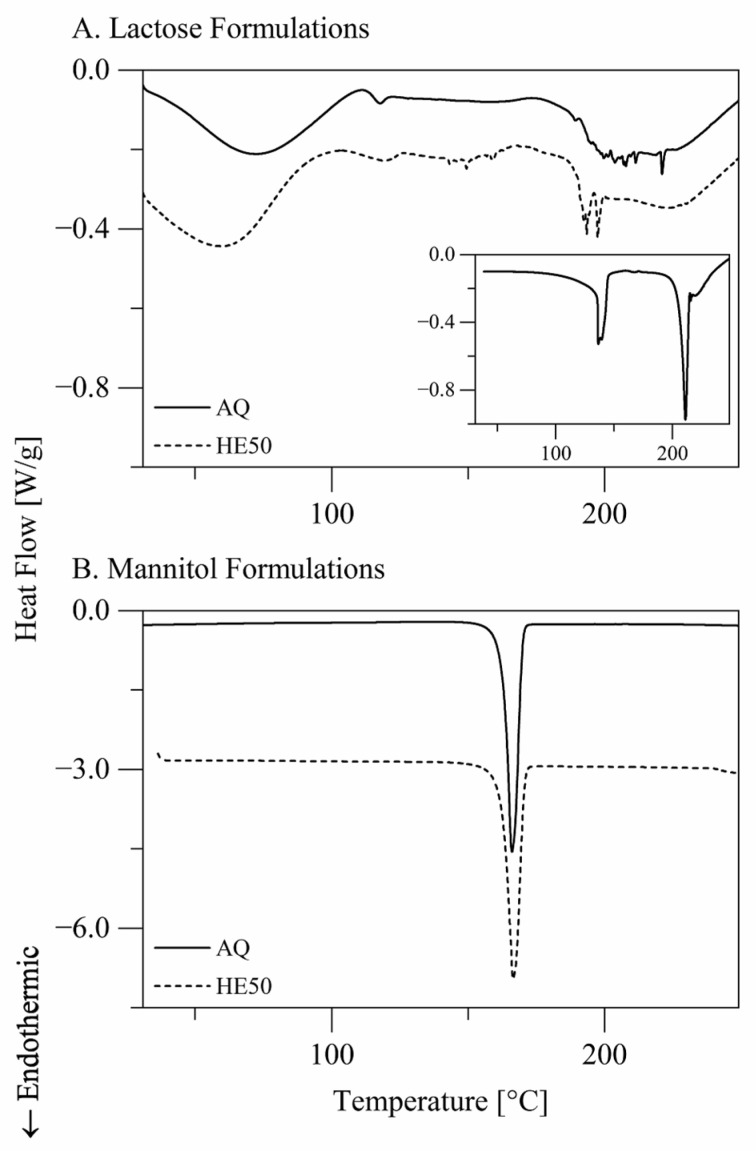
Differential scanning calorimetry thermograms of nano spray-dried (**A**) lactose and (**B**) mannitol powder materials. Presented data are for materials prepared by spray drying of aqueous (AQ) or hydroethanolic (HE50) solutions with a solute concentration of 3.00% *w*/*w* at an inlet temperature of 80 °C. Spray-dried lactose materials were amorphous. The insert of panel A presents data of crystalline α-lactose monohydrate material for comparison. Spray-dried mannitol materials were in the α/β crystalline form.

Differential scanning calorimetry (DSC) thermograms of selected nano spray-dried lactose and mannitol materials are presented in Figure 3. Spray-dried lactose materials were amorphous. The glass transition of lactose could be detected by DSC for some samples. The glass transition onset ($T_{g,\mathrm{onset}}$) of lactose was 112.85 ± 0.38 °C when spray dried from aqueous solutions and 106.19 ± 0.82 °C when spray dried from solutions comprising 50.0% *w*/*w* ethanol. The difference is a result of interference with the transition corresponding to water evaporation. Spray-dried mannitol materials were in the α/β crystalline form, which exhibited a melting transition with $T_{m,\mathrm{peak}}=$ 165.91 ± 0.50 °C when spray dried from aqueous solutions and $T_{m,\mathrm{peak}}=$ 166.09 ± 0.90 °C when spray dried from solutions comprising 50.0% *w*/*w* ethanol.

### 3.1. Spray Generation

Optimization of spray flow in the Nano Spray Dryer B-90 is one key to a successful process and yield optimization. The spray throughput is determined by the spray mesh pore size [5,15], the mesh vibration frequency, the flow rate of the spray solution through the spray head (pump speed), the spray solution viscosity [14,15,28], and the spray solution surface tension [14]. In the current study, for both lactose and mannitol, the spray rate was independent of the inlet temperature and the solute concentration in the spray solution (three-way analysis of variance: *p* > 0.1). However, the spray rate was lower with hydroethanolic solutions than with aqueous solutions (three-way analysis of variance and Tukey’s test: *p* < 0.01). This can be attributed to the relatively higher viscosities of the hydroethanolic solutions (Table 2).

In the current study, poor spray flow precluded spray drying of mannitol solutions in ultrapure water. The addition of hydrochloric acid at a low concentration of 0.02% *w*/*w* improved the spray flow for mannitol solutions. Hydrochloric acid is widely used to adjust the pH of inhalation formulations [29,30]. The use of hydrochloric acid was inspired by earlier experimental data [9,10], where terbinafine hydrochloride was co-spray dried with mannitol. In these two studies, hydrochloric acid was used for a different purpose: to maintain dissolution of terbinafine hydrochloride. The improvement of the spray flow of mannitol solutions here by addition of hydrochloric acid might be underlain by influences of hydrochloric acid on the surface tension of the spray solution, the conductivity of the spray solution, or the crystallization behavior of mannitol. Hydrochloric acid reduces the surface tension of water [31], but the effect estimated for the concentration used here is negligible. The conductivity of the spray solution is a possible determinant of the vibrating-mesh atomizer performance. The poor spray flow of mannitol solutions can be also caused by crystallization of mannitol on the surface of the vibrating mesh. The gas flow turbulence around the spray head sometimes draws spray droplets back to dry and deposit on the spray nozzle. Mannitol deposits, being crystalline and sticky, may have blocked the mesh pores or blocked mesh vibration. Nozzle deposits [14] and failure of the vibrating-mesh atomizer of the Nano Spray Dryer B-90 due to membrane blockage [18] have been earlier reported. We postulate that the improvement of the spray flow of mannitol solutions by addition of hydrochloric acid can be underlain by an influence of hydrochloric acid on the crystallization behavior of mannitol over the surface of the vibrating mesh. The improvement of the spray flow of mannitol solutions here by addition of hydrochloric acid is worth further investigation.

The spray rate did not here influence the yield or the moisture content (the loss on drying) of spray-dried lactose materials (aqueous solutions; three-way analysis of variance: *p* > 0.1). However, as mentioned earlier, the gas flow turbulence around the spray head sometimes drew spray droplets back to dry and deposit on the spray nozzle. The deposits ultimately fall over and usually end up on the surface of the inner high-voltage star electrode. Nozzle deposits lead to yield loss. Furthermore, the deposits sometimes contaminate the spray-dried yield deposited on the surface of the collection electrode. Nozzle deposits should thus be minimized or avoided. We used the amount of the powder material deposited on the high-voltage star electrode as a measure of nozzle deposits. The data of nano spray drying of aqueous lactose solutions suggest that nozzle deposits could be controlled by keeping the spray rate below 45 g/h. Nozzle deposits also decreased as the concentration of lactose in the spray solution increased. As the concentration of lactose in the spray solution increased from 1.5 to 3.0% *w*/*w*, nozzle deposits decreased from 9.2 ± 7.2% *w*/*w* to 2.1 ± 1.8% *w*/*w* with the aqueous spray solvent and from 7.9 ± 1.1% *w*/*w* to 1.6 ± 0.4% *w*/*w* with the hydroethanolic spray solvents (fractions of the amount of the solute dissolved in the spray solution; three-way analysis of variance and Tukey’s test: *p* < 0.01). Nozzle deposits were for mannitol consistently below 2.0% *w*/*w*, suggesting dependence on the material and/or the spray solution properties. Besides, a proportion of the spray-dried powder material sometimes deposited early on the surface of the glass cylinder (the drying chamber) before reaching the collection electrode. The data of nano spray drying of aqueous lactose solutions suggest that the amount of the powder material deposited on the glass cylinder could be reduced by increasing the spray rate. Increasing the spray rate from less than 25 g/h to more than 45 g/h reduced the amount of the powder material deposited on the glass cylinder from 15.2 ± 1.3% *w*/*w* to 7.0 ± 3.2% *w*/*w* (fractions of the amount of the solute dissolved in the spray solution; Tukey’s test: *p* < 0.001).

### 3.2. Temperature Profile

The temperature profile in the drying chamber of the Nano Spray Dryer is principally governed by the inlet temperature, the drying gas flow rate, and the spray rate [5]. In the current study, where the feed solution was kept at room temperature, the spray head temperature increased with both the inlet temperature and the spray rate. The increase in the spray head temperature with the spray rate is caused by heat generated due to mesh vibration. A multiple linear relationship well described the dependence:

(1) $$T_{\mathrm{SHead}}=\alpha +\beta T_{\mathrm{inlet}}+\gamma S\;.$$

**Figure 4:**
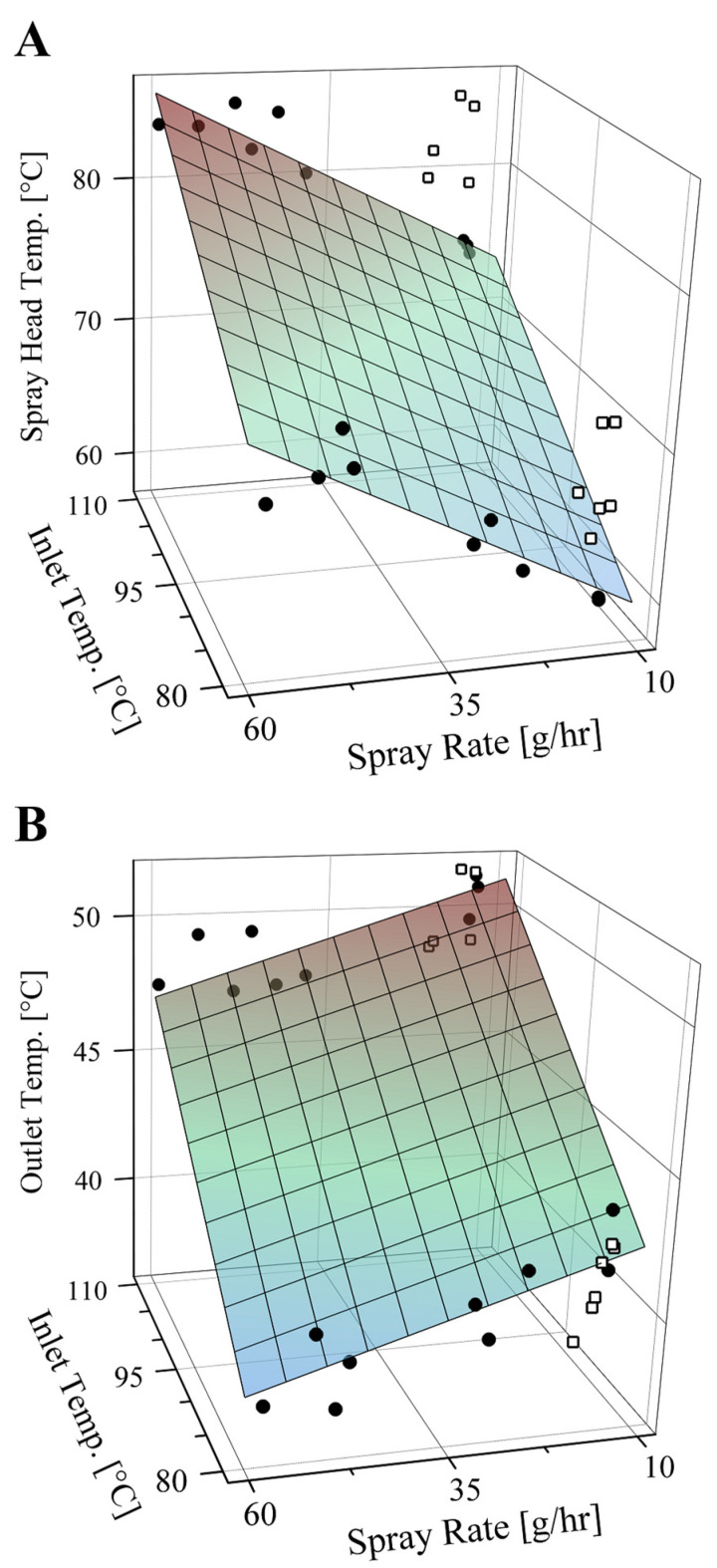
The dependence of the spray head temperature (panel (**A**)) and the outlet temperature (panel (**B**)) in the Nano Spray Dryer B-90 on the inlet temperature and the spray rate. The surface plots were simulated using multiple linear relationships derived from data of spray drying of aqueous lactose solutions. Filled circles represent actual data of aqueous lactose solutions. Open squares represent actual data of hydroethanolic lactose solutions for comparison.

$T_{\mathrm{inlet}}$ and $T_{\mathrm{SHead}}$ are the inlet and the spray head temperatures in °C. S is the spray rate in g/h. Multiple linear regression of data measured in the current study with aqueous lactose solutions gave α= 20.3 ± 2.7, β= 0.461 ± 0.027, and γ= 0.251 ± 0.025 (fitted coefficients ± standard errors, RMSE = 1.72 °C, $R^{2}=$ 0.964). The relationship is presented in Figure 4A. The spray head temperatures were higher with hydroethanolic solutions than with aqueous solutions of lactose (Figure 4A). The difference amounted to 5.7 ± 0.4 °C and 11.1 ± 0.5 °C (means ± standard errors; Tukey’s test: *p* < 0.001) for solutions comprising 15% *w*/*w* and 50% *w*/*w* ethanol, respectively. This finding can be explained by (i) the higher viscosities of the hydroethanolic solutions and hence poorer flow through the spray head and (ii) the lower specific heat capacities of the hydroethanolic solutions. The high temperature of the spray head sometimes demands cooling of the spray solution, especially for thermolabile materials and with volatile spray solvents. Evaporation of the solvent in the spray solution bottle can lead to solute precipitation.

The outlet temperature increases with the inlet temperature and the drying gas flow rate and decreases as the spray rate increases. Based on literature data, Arpagaus et al. [5] demonstrated linear dependence of the outlet temperature on the inlet temperature in the Nano Spray Dryer B-90: $T_{\mathrm{outlet}}=a+bT_{\mathrm{inlet}}$, where $T_{\mathrm{inlet}}$ and $T_{\mathrm{outlet}}$ are the inlet and the outlet temperatures in °C. From the literature data, Arpagaus et al. [5] calculated a= 14.31 and b= 0.333 for aqueous spray solutions. Linear fitting of data measured in the current study with aqueous lactose solutions gave a= 17.7 ± 2.6 and b= 0.285 ± 0.027 (fitted coefficients ± standard errors, $R^{2}=$ 0.872). The coefficients are close to those calculated by Arpagaus et al. [5]. In the current study, we used multiple linear regression to consider the influence of the spray rate as well on the outlet temperature.

(2) $$T_{\mathrm{outlet}}=\alpha +\beta T_{\mathrm{inlet}}+\gamma S\;\;.$$

Multiple linear regression of data measured in the current study with aqueous lactose solutions gave α= 20.1 ± 1.8, β= 0.289 ± 0.018, and γ= −0.079 ± 0.016 (fitted coefficients ± standard errors, RMSE = 1.12 °C, $R^{2}=$ 0.950). The relationship is presented in Figure 4B. The outlet temperature was independent of the spray solvent and the solute concentration in the spray solution (three-way analysis of variance: *p* > 0.05). The outlet temperatures with hydroethanolic lactose solutions were well predicted by the relationship derived from data of aqueous solutions (RMSE = 1.57 °C, Figure 4B).

#### 3.2.1. Yield

Attempts in the literature [11,12,14,17,18,28] to study the influence of the inlet temperature on the yield of the Nano Spray Dryer B-90 do not reveal a solid, consistent relationship. The variability of the Nano Spray Dryer B-90 yield [9,10,12] hinders detection of possible general dependencies. The variability is caused mainly by gas flow turbulence around the spray head (see Section 3.1 and Section 3.3.1). In the current study, the inlet temperature did not influence the spray drying yield for lactose and mannitol with aqueous and hydroethanolic solvents (three-way analysis of variance: *p* > 0.05).

#### 3.2.2. Moisture Content

The temperature profile and the gas flow in the drying chamber of the Nano Spray Dryer determines the thermal load and the drying kinetics. To achieve a final product with a low residual moisture content, the inlet temperature has to be set as high as possible and the difference between the inlet and the outlet temperatures has to be as small as possible [32]. Although increasing the inlet temperature is expected to reduce the moisture content of the spray-dried material, the thermal efficiency and the small size of spray droplets in the Nano Spray Dryer apparently neutralize such dependency. For example, Schmid et al. [14] did not detect an evident influence for the inlet temperature (60 to 120 °C) on the moisture content of trehalose and mannitol materials spray-dried by the Nano Spray Dryer B-90.

In the current study, the inlet temperature generally reduced the moisture content (the loss on drying) of spray-dried lactose materials. The residual moisture content of spray-dried lactose materials generally amounted to 4.7 ± 0.8% *w*/*w*. Increasing the inlet temperature from 80 °C to 110 °C reduced the moisture content from 5.3 ± 0.9% *w*/*w* to 4.2 ± 0.5% *w*/*w* with the aqueous solvent and from 4.8 ± 0.2% *w*/*w* to 4.2 ± 0.6% *w*/*w* with the hydroethanolic solvents (three-way analysis of variance and Tukey’s test: *p* < 0.05). On the other hand, the residual moisture content of spray-dried mannitol materials generally amounted to 1.2 ± 0.3% *w*/*w* and was independent of the inlet temperature (three-way analysis of variance: *p* > 0.1). The difference in the residual moisture content between spray-dried lactose and mannitol materials reflects the difference in crystallinity (Figure 3).

#### 3.2.3. Particle Size Distribution

**Figure 5:**
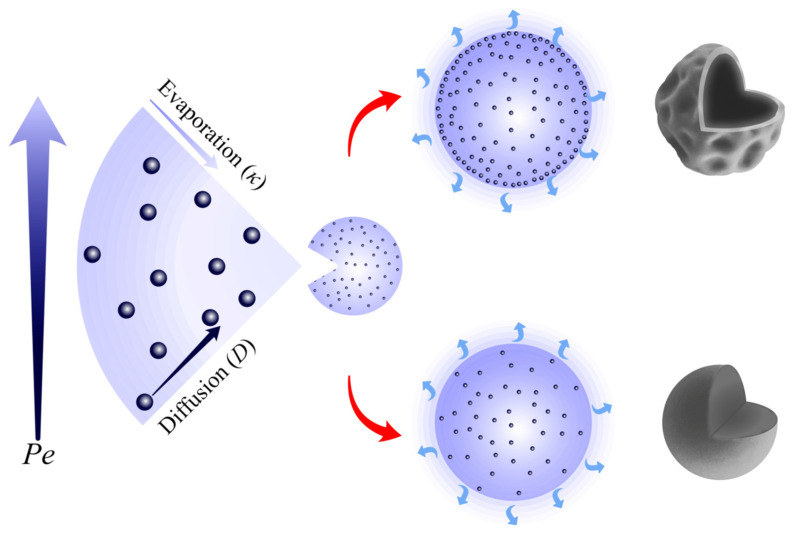
An illustration of the Péclet number concept. The higher the Péclet number, the higher the rate of radial shrinkage of spray droplets relative to the rate of inward diffusion of solute molecules.

The influence of the drying temperature on the structure of spray-dried particles is often explored in light of the Péclet number concept (Figure 5). The Péclet number illustrates the interplay between radial shrinkage of spray droplets and inward diffusion of solute molecules [1,2,3]:

(3) $$Pe_{i}=\kappa /8D_{i}\;.$$

κ is the solvent evaporation rate: $\kappa =d_{0}{}^{2}/\tau_{D}$, where $d_{0}$ is the droplet diameter and $\tau_{D}$ the droplet drying time. $D_{i}$ is the diffusion coefficient of the solute i in the spray solution. The higher the Péclet number, the higher the rate of radial shrinkage of spray droplets relative to the rate of inward diffusion of solute molecules. A higher Péclet number corresponds to particles with relatively larger size and lower density, i.e., hollower structure and thinner shells. Vehring et al. [33] demonstrated that increasing the drying rate by increasing the drying temperature in a well-controlled spray drying environment leads to relatively larger particles with relatively lower density. Spray-dried lactose and mannitol particles were here obviously hollow (Figure 2), suggesting high evaporation rates and $Pe_{i}>1$.

The Péclet number concept is generally useful. It satisfactorily explains the spray-drying behavior of large molecular weight compounds, e.g., proteins and nanoparticle aggregates [33,34]. It is, however, simplified since it assumes the evaporation rate of the solvent and the diffusion coefficient of the solute are constants. It does not account for changes in the droplet temperature or changes in the composition of non-azeotropic cosolvent systems during evaporation. Computational fluid dynamics simulations could reveal that the drying rate in the Nano Spray Dryer B-90 can vary during a single droplet’s lifetime by several orders of magnitude and vary among droplets in a single spray drying experiment with coefficients of variation in the range of 40–230% [19]. The Péclet number concept does not consider forces such as surface activity, which can drive solute molecules towards surfaces of drying droplets. It does not consider changes in the droplet viscosity and the solute diffusion coefficient taking place as the solute starts to crystallize/precipitate.

In the current study, the inlet temperature did not influence the particle size distribution of spray-dried lactose materials. All descriptors ($D_{\mathrm{mean}}$, $D_{10}$, $D_{50}$, $D_{90}$, and Span) of the particle size distribution (number-weighted and volume-weighted) of spray-dried lactose materials were independent of the inlet temperature (three-way analysis of variance: *p* > 0.1). The number-weighted span values were around 2.0 (Table 3), indicating narrow size distributions achieved by the vibrating-mesh technology. Analysis of the number-weighted particle size distributions of spray-dried mannitol materials did not reveal temperature dependence (three-way analysis of variance: *p* > 0.1). Analysis of the volume-weighted particle size distributions of spray-dried mannitol materials highlighted, however, that the median particle diameter marginally increased with the increase in the inlet temperature from 80 to 110 °C (three-way analysis of variance: *p* > 0.05; Tukey’s test: *p* < 0.05, 95% CI = 0.2–2.6 μm).

It was not surprising to find that the drying temperature in the Nano Spray Dryer B-90 has almost no influence on the size of spray-dried particles. The conclusion agrees with literature reports of no or minor relationship [11,12,16,17,18]. Indeed, the inlet temperature range in the Nano Spray Dryer B-90 is narrow. In light of the Péclet number concept, it should also be noted that both the evaporation rate and the diffusion coefficient are temperature dependent, especially when the spray solution is heated as it is circulated through the spray head. The temperature dependence of the evaporation rate can be illustrated by measurements of Vehring et al. [33], who measured the evaporation rate of pure water droplets with ~16 μm initial diameter to increase approximately from 2.7 to 6.0 μm^2^/ms as the temperature increases from 50 to 100 °C [33]. The temperature dependence of the diffusion coefficient can be illustrated by Stokes–Einstein equation. Accordingly, $D_{i}\propto T/\eta$, where T is the absolute temperature and η is the viscosity of the solvent. The viscosity of liquid water decreases from 0.547 to 0.283 mPa⋅s as the temperature increases from 50 to 99.6 °C [35].

### 3.3. Spray Solvent

#### 3.3.1. Yield

In the current study, the spray solvent influenced the yield of spray drying of lactose. Using a hydroethanolic rather than an aqueous spray solvent for lactose increased the yield from 66.6 ± 3.8% *w*/*w* to 75.9 ± 2.7% *w*/*w* (spray rate < 25 g/h; three-way analysis of variance and Tukey’s test: *p* < 0.001). However, the yield was independent of the concentration of ethanol in the spray solution (50.0 vs. 15.0% *w*/*w*; Tukey’s test: *p* > 0.1). The yield of spray drying of mannitol was independent of the spray solvent (three-way analysis of variance and Tukey’s test: *p* > 0.1).

We have recently [9] found that the yield of nano spray drying of terbinafine hydrochloride with either lactose or mannitol considerably increased when water was replaced with a hydroethanolic mixture (50.5% *w*/*w* ethanol in water) as a spray solvent. The yield of nano spray drying of a β-galactosidase-trehalose material was also reported to increase when water was replaced with 20% *v/v* ethanol in water as a spray solvent [17]. The influence was attributed to the low spray rate associated with the hydroethanolic solvent. However, the comparison here for lactose is based on experiments with similar spray rates. If the powder material deposited on the surface of the collection electrode and the powder material deposited on the glass cylinder are summed up, the total yield turns out to be independent of the spray solvent (three-way analysis of variance and Tukey’s test: *p* > 0.1). The influence of the spray solvent on the yield is apparently underlain by the spray flow turbulence rather than by the spray rate. Developing a nano spray drying procedure should consider optimizing the spray flow, for example, using surface-active additives, e.g., polysorbate 20.

#### 3.3.2. Moisture Content

The moisture contents (the losses on drying) of spray-dried lactose and mannitol materials were independent of the spray solvent (Table 3; three-way analysis of variance: *p* > 0.05). Besides the loss on drying, we considered the moisture evaporation enthalpy measured by differential scanning calorimetry (DSC) at T= 30–110 °C (Figure 3) as a measure of the moisture content. The water evaporation enthalpy was smaller for lactose materials spray dried from solutions comprising 50.0% *w*/*w* ethanol than for lactose materials spray dried from aqueous solutions (Table 3; Tukey’s test: *p* < 0.01). DSC was more sensitive to the moisture content than the loss on drying. The low moisture contents of spray-dried mannitol materials were undetectable by DSC.

#### 3.3.3. Particle Size Distribution

The size of spray droplets generated by the Nano Spray Dryer vibrating-mesh atomizer is mainly determined by the spray mesh pore size [11,12,14,15,16,17]. The size of spray droplets generated by a similar vibrating-mesh atomizer was also found to decrease as the viscosity of the spray solution increases [36]. The particle size of a nano spray-dried β-galactosidase-trehalose material was reported to decrease when water was replaced with 20% *v/v* ethanol in water as a spray solvent [17]. This finding could be attributed to a possible influence on the size of spray droplets: the higher viscosity or the lower surface tension of the hydroethanolic solution might have resulted in smaller spray droplets.

**Figure 6:**
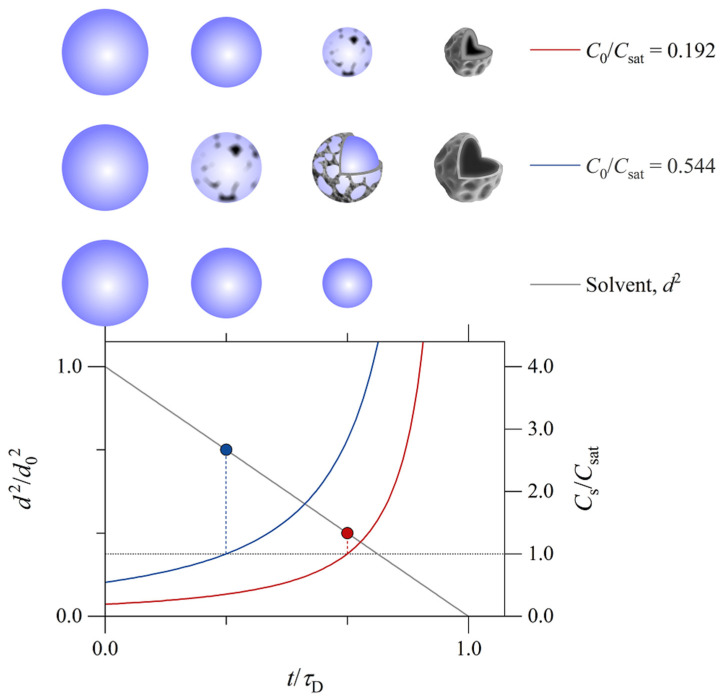
An illustration of the time required for the solute to reach saturation at the surface of a drying droplet. The temporal evolution of the droplet surface area (black line, left axis) was calculated assuming that the droplet surface area decreases linearly with time. The droplet surface area is presented as a fraction of the initial droplet surface area, where d is the droplet diameter and $d_{0}$ the initial droplet diameter. The temporal evolution of the solute concentration at the surface of the drying droplet was calculated using Equation (4) (red and blue lines, right axis). The solute concentration at the surface of the drying droplet, $C_{s}$, is presented as a fraction of the saturation solubility of the solute in the spray droplet/solution, $C_{\mathrm{sat}}$. The initial concentration of the solute in the spray droplet/solution, $C_{0}$, was set to 0.192 $C_{\mathrm{sat}}$ (red) and 0.544 $C_{\mathrm{sat}}$ (blue). Particle formation is illustrated for the two situations. The dotted horizontal line corresponds to surface saturation, i.e., $C_{s}=C_{\mathrm{sat}}$. The time, t, is presented as a fraction of the droplet drying time, $\tau_{D}$.

The influence of the spray solvent on the size of spray-dried particles is also mediated by the time, $\tau_{\mathrm{sat}}$, required for the solute to reach saturation at the surface of a drying droplet (Figure 6). The concentration of a solute *i* at the surface of a drying droplet at time t can be expressed by Equation (4):

(4) $$C_{s,i}=C_{0,i}E_{i}{\left(1-\frac{t}{\tau_{D}}\right)}^{-\frac{3}{2}}.$$

$C_{0,i}$ is the initial concentration of the solute in the spray droplet. $E_{i}=C_{s,i}/C_{\mathrm{av},i}$ is the surface enrichment, defined as the ratio between the surface concentration and the average concentration of the solute in the droplet. $\tau_{D}=d_{0}{}^{2}/\kappa$ is the droplet drying time, where $d_{0}$ is the droplet diameter and κ the solvent evaporation rate. The time, $\tau_{\mathrm{sat}}$, required for the solute *i* to reach saturation at the surface of the drying droplet can then be expressed by Equation (5) [1,2,37]:

(5) $$\tau_{\mathrm{sat},i}=\frac{d_{0}{}^{2}}{\kappa }\left[1-{\left(E_{i}\frac{C_{0,i}}{C_{\mathrm{sat},i}}\right)}^{\frac{2}{3}}\right].$$

$C_{\mathrm{sat},i}$ is the saturation solubility of the solute in the spray droplet. $C_{0,i}/C_{\mathrm{sat},i}$ is the initial degree of saturation. Assuming that the geometric diameter of the spray-dried particle is equal to the droplet diameter when the solute *i* reaches surface saturation, the geometric particle diameter, $d_{g}$, can be expressed by Equation (6):

(6) $$d_{g}=d_{0}{\left(E_{i}\frac{C_{0,i}}{C_{\mathrm{sat},i}}\right)}^{\frac{1}{3}}.$$

The smaller the saturation solubility, the shorter the time required for the solute to reach saturation at the surface of the drying droplet, the larger the geometric diameter of the spray-dried particle, and the smaller the density of the particle. The influence of the spray solvent on the size of spray-dried particles (data reported in [38,39], as examples) is often better explained by the associated change in the saturation solubility of the solute in spray droplets (Equation (6)) than by the associated change in the Péclet number (Equation (3)). Indeed, the associated change in the degree of saturation is often greater than the effective change in the evaporation rate.

In the current study, lactose materials spray dried from hydroethanolic solutions exhibited marginally smaller particle size than lactose materials spray dried from aqueous solutions (Table 3). Descriptors ($D_{\mathrm{mean}}$, $D_{10}$, $D_{50}$, and $D_{90}$) of the number-weighted particle size distribution of spray-dried lactose materials were smaller with hydroethanolic solutions than with aqueous solutions (three-way analysis of variance and Tukey’s test: *p* < 0.05). The number-weighted particle size distribution was, however, independent of the concentration of ethanol in the spray solution (50.0 vs. 15.0% *w*/*w*; Tukey’s test: *p* > 0.1). Analysis of the volume-weighted particle size distributions showed less dependence on the spray solvent: the median particle diameter was not statistically different with hydroethanolic solutions than with aqueous solutions (three-way analysis of variance and Tukey’s test: *p* > 0.05). The particle size of spray-dried mannitol materials exhibited, similarly, in terms of the number-weighted but not in terms of the volume-weighted particle size distribution, marginal dependence on the spray solvent (Table 3). The particle size data suggest that hydroethanolic solutions were associated with somewhat smaller spray droplets than aqueous solutions. This was obvious despite that the influence of the spray solvent on the size of spray droplets was here opposed by the influence of the spray solvent on the saturation solubilities of lactose and mannitol in spray droplets (Table 1). It is noteworthy that the current study is limited to hydrophilic materials. Here, we did not study materials which are more soluble in the hydroethanolic spray solvents than in the aqueous spray solvent. For such materials, the influences of the hydroethanolic spray solvent on the size of spray droplets and on the saturation solubility of the material in spray droplets are both expected to reduce the geometric diameter of spray-dried particles.

In an earlier study conducted in our labs [9], we used nano spray drying to prepare inhalable particles of terbinafine hydrochloride with either lactose or mannitol. Using a hydroethanolic mixture (50.5% *w*/*w* ethanol in water) as a spray solvent resulted in considerably smaller particles than with water as a spray solvent. The Péclet number concept could not explain the observation: the hydroethanolic solvent had a faster evaporation rate and was more viscous than water, i.e., the hydroethanolic solvent was associated with a relatively higher Péclet number and was expected to result in relatively larger particles. Similarly, the observation is opposite to what can be predicted from the saturation solubility of the major solute (lactose or mannitol, 90% *w*/*w*) in spray droplets. We presumed that the observation was underlain by the influence of the spray solvent viscosity on the size of spray droplets. The influence of the spray solvent on the size of spray-dried particles was in our earlier study more considerable than observed here. It is plausible to conclude that terbinafine hydrochloride contributed to the observation of our earlier study. In contrast to lactose and mannitol, terbinafine hydrochloride was indeed more soluble in the hydroethanolic than in the aqueous spray solvent.

It is noteworthy that the discussion here addressed only the geometric particle size. The aerodynamic diameter is the particle property relevant to pulmonary drug delivery. The saturation solubility of the solute in spray droplets influences the geometric diameter and the aerodynamic diameter of spray-dried particles differently. This is because the change in the geometric particle diameter is associated with a change in the particle density. The relationship between the geometric diameter and the aerodynamic diameter of a particle is expressed by Equation (7):

(7) $$d_{a}=d_{g}{\left(\frac{\rho_{p}}{\chi }\right)}^{\frac{1}{2}}.$$

$\rho_{p}$ is the particle density and χ the dynamic shape factor. χ= 1 for spherical particles. Combining Equation (6) and Equation (7) gives Equation (8):

(8) $$d_{a}=d_{0}{\left(\frac{C_{0,i}}{\chi }\right)}^{\frac{1}{3}}{\left(\frac{C_{\mathrm{sat},i}}{\chi E_{i}}\right)}^{\frac{1}{6}}.$$

### 3.4. Solute Concentration

#### 3.4.1. Yield

Literature data [11,12,20,28] regarding the influence of the solute concentration in the spray solution on the yield of the Nano Spray Dryer B-90 does not reveal a solid, consistent relationship. In the current study, the yield of spray drying of lactose with aqueous and hydroethanolic solutions was independent of the concentration of lactose in the spray solution (three-way analysis of variance: *p* > 0.1). On the other hand, the yield of spray drying of mannitol increased from 63.1 ± 1.5% *w*/*w* to 69.0 ± 1.6% *w*/*w* (three-way analysis of variance and Tukey’s test: *p* < 0.01) as the concentration of mannitol in the spray solution increased from 1.5 to 3.0% *w*/*w*.

#### 3.4.2. Moisture Content

Generally, the moisture content (the loss on drying) of spray-dried materials was independent of the solute concentrations in the spray solution (three-way analysis of variance: *p* > 0.1), for both lactose and mannitol, with aqueous and with hydroethanolic solutions. Remarkably, spray drying of 3.0% *w*/*w* lactose solutions at an inlet temperature of 80 °C resulted in powder materials with higher water evaporation enthalpies than other corresponding spray-dried lactose materials (i.e., compared to 1.5% *w*/*w* at 80 °C, 1.5% *w*/*w* at 110 °C, and 3.0% *w*/*w* at 110 °C with the same spray solvent; *p* < 0.01, Tukey’s test). This finding suggests an interaction with the influence of the inlet temperature on the product moisture content.

#### 3.4.3. Particle Size Distribution

The particle size of a spray-dried material typically increases with the solute concentration in the spray solution [11,12,16,20,40]. This is expressed by Equation (6). The higher the solute concentration in the spray solution, the shorter the time required for the solute to reach saturation at surfaces of drying droplets and the larger the geometric diameter of spray-dried particles. The solute concentration in the spray solution influences the geometric diameter and the aerodynamic diameter of spray-dried particles similarly (Equations (6) and (8)).

In the current study, increasing the solute concentration in the spray solution from 1.5 to 3.0% *w*/*w* was associated with a marginal increase in the size of spray-dried particles. The mean difference in the number-weighted median particle diameter amounted to 0.2 ± 0.1 μm for lactose and 0.3 ± 0.0 μm for mannitol (means ± standard errors; Tukey’s test: *p* < 0.01). The differences were less significant when the data were volume weighted. The small differences agree with the theoretical model, where the geometric diameter of spray-dried particles is proportional to the cube root of the solute concentration in the spray solution.

## 4. Conclusions

In the present study, the process parameters of the Büchi Nano Spray Dryer B-90 were investigated following a factorial experimental design using lactose and mannitol as model materials. The interplay between the process parameters with regard to optimization of the spray dryer performance and particle properties is summarized in Table 4.

The results can be summarized as follows:

- Optimization of spray flow in the Nano Spray Dryer B-90 is one key to a successful process and yield optimization. The addition of hydrochloric acid at a low concentration of 0.02% *w*/*w* here improved the spray performance for mannitol solutions. Possibly, hydrochloric acid influenced the crystallization behavior of mannitol in a way that prevented blockage of the spray mesh pores or the spray mesh vibration by mannitol crystals. The observation is worth further investigation. Using additives that modulate the crystallization behavior of the material to be spray dried is suggested as an approach to optimize spray generation by the vibrating-mesh atomizer. An alternative is the use of surface-active additives, e.g., polysorbate 20.
- Powder yields (2.0 g batches) were generally 71% *w*/*w* for lactose and 66% *w*/*w* for mannitol. Using a hydroethanolic spray solvent increased the yield to 76% *w*/*w* for lactose. The increase is attributed to lower turbulence around the spray head. The influence of the spray solvent on the flow of spray droplets can be considered as an approach for yield optimization.
- Powder analysis revealed that lactose and mannitol particles were spherical. Spray-dried lactose materials were amorphous, whereas mannitol materials were crystalline.
- Particle size was influenced by the spray solvent. The spray solvent viscosity and surface tension and the time required for the solute to reach saturation at surfaces of drying droplets contribute to the final particle size.

The analysis lends itself as guidance on optimization of particle engineering by nano spray drying.

## Figures and Tables

**Table 1:** Properties of materials used in the current study.

Property ^a^	Lactose	Mannitol
MW [g/mol]	342.30	182.17
$T_{g,\mathrm{onset}}$ [°C]	102 [22]	10.7 [23]
$T_{m}$ [°C]	214–219 (α, peak) [24], 236 (β, peak) [25]	150–158 (δ, onset) [26], 166 (α/β, onset) [26]
$S_{W}$ [% *w*/*w*]	29 (37 °C) [27]	23.8 (37 °C) [27]
$S_{\mathrm{HE}}$ [% *w*/*w*]	3.5 (37 °C) [27]	6.0 (37 °C) [27]

^a^ MW is the molecular weight (anhydrous basis). $T_{g,\mathrm{onset}}$ is the glass transition temperature (onset). $T_{m}$ is the melting transition temperature (onset or peak). When applicable, information about melting transitions of different crystalline forms is given. $S_{W}$ is the solubility in water. $S_{\mathrm{HE}}$ is the solubility in a 50% *w*/*w* ethanol-in-water mixture.

**Table 2:** Design of nano spray drying experiments.

Set of Experiments	1	2	3	4	5
	Lactose-AQ	Lactose-HE15	Lactose-HE50	Mannitol-AQ	Mannitol-HE50
Solute	Lactose	Lactose	Lactose	Mannitol	Mannitol
Concentration of solute [% *w*/*w*]	1.5, 3.0	1.5, 3.0	1.5, 3.0	1.5, 3.0	1.5, 3.0
Solvent	Aqueous	Hydroethanolic	Hydroethanolic	Aqueous	Hydroethanolic
Concentration of ethanol [% *w*/*w*]	0.0	15.0	50.0	0.0 ^a^	50.0 ^a^
Solution density, 40 °C [g/cm^3^]	0.998–1.002	0.970–0.979	0.904–0.906	0.997–1.001	0.901–0.904
Solution viscosity, 40 °C [mPa⋅s]	0.64–0.69	1.19–1.21	1.69–1.76	0.71–0.82	1.65–1.69
Spray intensity [%]	50–100	75–85	100	75	100
Spray rate [g/h]	14–60	14–21	14–17	24–28	9–15
Inlet temperature [°C]	80, 110	80, 110	80, 110	80, 110	80, 110
Air flow rate [L/min]	135	135	135	135	135
Number of experiments (N)	18	7	4	4	4

^a^ Hydrochloric acid was added to a concentration of 0.02% *w*/*w* to all mannitol spray solutions.

**Table 3:** Characteristics of nano spray-dried powder materials.

Set of Experiments	1	2	3	4	5
	Lactose-AQ	Lactose-HE15	Lactose-HE50	Mannitol-AQ	Mannitol-HE50
Yield [% *w*/*w*]	68.2 ± 6.6	76.1 ± 2.8	75.4 ± 2.9	65.7 ± 3.5	66.4 ± 4.0
Glass cylinder [% *w*/*w*]	11.1 ± 4.5	7.2 ± 3.7	7.0 ± 2.4	11.1 ± 3.4	8.8 ± 2.0
Nozzle deposits [% *w*/*w*]	5.3 ± 6.0	4.6 ± 3.6	4.3 ± 3.5	0.8 ± 1.0	0.9 ± 0.6
Loss on drying [% *w*/*w*]	4.7 ± 0.9	4.5 ± 0.6	4.5 ± 0.5	1.0 ± 0.1	1.3 ± 0.2
$D_{N,\mathrm{mean}}$ [μm]	2.2 ± 0.3 ^a^	1.9 ± 0.1	1.9 ± 0.2	2.3 ± 0.2	2.0 ± 0.3
$D_{N,10}$ [μm]	0.7 ± 0.1 ^a^	0.6 ± 0.0	0.7 ± 0.0	0.7 ± 0.0	0.6 ± 0.0
$D_{N,50}$ [μm]	1.8 ± 0.2 ^a^	1.5 ± 0.1	1.5 ± 0.2	1.9 ± 0.1	1.5 ± 0.2
$D_{N,90}$ [μm]	4.3 ± 0.8 ^a^	3.5 ± 0.3	3.6 ± 0.4	4.6 ± 0.5	4.0 ± 1.1
${\mathrm{Span}}_{N}$	2.0 ± 0.3 ^a^	1.9 ± 0.1	1.9 ± 0.1	2.0 ± 0.1	2.2 ± 0.5
$D_{V,\mathrm{mean}}$ [μm]	5.3 ± 0.7 ^a^	4.5 ± 0.5	4.7 ± 0.5	5.7 ± 0.7	5.5 ± 1.9
$D_{V,10}$ [μm]	2.6 ± 0.5 ^a^	2.1 ± 0.2	2.2 ± 0.2	2.8 ± 0.2	2.6 ± 0.9
$D_{V,50}$ [μm]	5.3 ± 0.9 ^a^	4.4 ± 0.7	4.7 ± 0.3	5.6 ± 0.6	5.6 ± 1.9
$D_{V,90}$ [μm]	8.0 ± 1.0 ^a^	6.9 ± 0.7	7.1 ± 0.7	8.6 ± 1.0	8.1 ± 2.7
${\mathrm{Span}}_{V}$	1.0 ± 0.1 ^a^	1.1 ± 0.1	1.0 ± 0.1	1.0 ± 0.1	1.0 ± 0.1
Enthalpy of water evap. [J/g]	66.2 ± 8.0 ^a^	61.8 ± 10.9	45.8 ± 13.8	ND *	ND *
$T_{g,\mathrm{onset}}$ [°C]	112.85 ± 0.38 ^a^	NM *	106.19 ± 0.82 ^b^	ND *	ND *
$T_{m,\mathrm{peak}}$ [°C]	ND *	ND *	ND *	165.91 ± 0.50	166.09 ± 0.90
Enthalpy of melting [J/g]	ND *	ND *	ND *	261.2 ± 13.4	261.7 ± 5.4

* ND = not detected; NM = not measured. ^a^ N= 3–5, spray rate < 25 g/h. ^b^ N= 2.

**Table 4:** The process parameters of the Büchi Nano Spray Dryer B-90 and their major influences on the spray dryer performance and product characteristics.

Process Parameter	Major Influence
Spray throughput	Nozzle deposits and yield loss
Inlet temperature	Moisture content of amorphous but not crystalline materials
Spray mesh	Particle size and spray throughput
Spray solvent	Particle size, density, and morphology mediated mainly by the influence of the solvent on the solubility of the solute and the time it requires to reach saturation at surfaces of drying droplets $d_{P}\propto {\left(1/C_{\mathrm{sat}}\right)}^{\frac{1}{3}}$
Solute concentration	Particle size mediated mainly by the influence of the concentration on the time the solute requires to reach saturation at surfaces of drying droplets $d_{P}\propto {\left(C_{0}\right)}^{\frac{1}{3}}$

## Data Availability

The data presented in this study are available in detail in Mendeley Data at https://doi.org/10.17632/twmxpnk2tx.1 (published on 8 March 2022).
